# A novel scoring system predicting survival benefits of palliative primary tumor resection for patients with unresectable metastatic colorectal cancer: A retrospective cohort study protocol

**DOI:** 10.1097/MD.0000000000017178

**Published:** 2019-09-13

**Authors:** Gaoyang Cao, Wei Zhou, Engeng Chen, Fei Wang, Li Chen, Min Chen, Wei Zhao, Jianbin Xu, Wei Zhang, Guolin Zhang, Xuefeng Huang, Zhangfa Song

**Affiliations:** a Department of Colorectal Surgery, Sir Run Run Shaw Hospital of Zhejiang University; b Zhejiang Province Key Laboratory of Biological Treatment, Hangzhou; c The Second Affiliated Hospital of Zhejiang University School of Medicine, Lanxi Hospital, China.

**Keywords:** metastatic colorectal cancer, novel scoring system, palliative primary tumor resection, prognosis, survival

## Abstract

The role of palliative primary tumor resection (PPTR) in improving survival in patients with synchronous unresectable metastatic colorectal cancer (mCRC) is controversial. In this study, we aimed to evaluate whether our novel scoring system could predict survival benefits of PPTR in mCRC patients.

In this retrospective cohort study consecutive patients with synchronous mCRC and unresectable metastases admitted to Sir Run Run Shaw Hospital between January 2005 and December 2013 were identified. A scoring system was established by the serum levels of carcinoembryonic antigen (CEA), cancer antigen 19-9 (CA19-9), neutrophil/lymphocyte ratio (NLR), and lactate dehydrogenase (LDH). Patients with scores of 0, 1–2, or 3–4 were considered as being in the low, intermediate, and high score group, respectively. Primary outcome was overall survival (OS).

A total of 138 eligible patients were included in the analysis, of whom 103 patients had undergone PPTR and 35 had not. The median OS of the PPTR group was better than that of the Non-PPTR group, with 26.2 and 18.9 months, respectively (*P* < .01). However, the subgroup of PPTR with a high score (3–4) showed no OS benefit (13.3 months) compared with that of the Non-PPTR group (18.9 months, *P* = .11). The subgroup of PPTR with a low score (52.1 months) or intermediate score (26.2 months) had better OS than that of the Non-PPTR group (*P* < .001, *P* = .017, respectively).

A novel scoring system composed of CEA, CA19-9, NLR, and LDH values is a feasible method to evaluate whether mCRC patients would benefit from PPTR. It might guide clinical decision making in selecting patients with unresectable mCRC for primary tumor resection.

## 1 Introduction

Colorectal cancer (CRC) is one of the leading causes of cancer-related death worldwide. The mortality rate and incidence of CRC in China in 2015 both rank fifth up to 191.0 and 376.3 (per 100,000), respectively.^[1]^ Approximately 22% to 25% of CRC patients have synchronous metastasis at the time of diagnosis.^[2,3]^ About 75% to 85% of these metastatic lesions are unresectable.^[4]^ Palliative primary tumor resection (PPTR) may be required in patients with obstruction, perforation, or bleeding, but whether the procedure should be done in asymptomatic patients is still being debated. Some studies support PPTR because it decreases the mortality and morbidity risk compared to that of emergency procedures.^[5]^ However, the development of new chemotherapeutic and molecular-targeting agents has also helped to well control the primary tumor and prolong the survival time.^[6–9]^ Whether PPTR has benefit of survival or improves the quality of life is still unclear. All published studies are retrospective in design and the conclusions are contradictory. Randomized controlled studies have not been reported yet.

Since there have been no randomized clinical trials (RCTs) on PPTR benefit for metastatic CRC (mCRC) patients thus far, another way to resolve the question is through an index or scoring system for screening patients who can benefit from PPTR and exclude those who may not. Recent researchers have reported on several cancer-related inflammation parameters that may play a key role in cancer development, progression, and metastasis leading to worse prognosis,^[10]^ such as the neutrophil/lymphocyte ratio (NLR), platelet/lymphocyte ratio (PLR), lymphocyte/monocyte ratio (LMR), and Glasgow prognostic score.^[11,12]^ NLR is one of the inflammation-based prognostic parameters that has been investigated in several types of cancer.^[13–16]^ In addition, studies have reported that patients with mCRC and a low level of carcinoembryonic antigen (CEA) may benefit from PPTR.^[17]^ In this study, we have attempted to identify additional serum markers to improve the accuracy of predicting which part of mCRC patients could benefit from PPTR. And we have proposed a novel prognostic scoring system involving the serum levels of CEA, NLR, cancer antigen 19-9 (CA19-9), and lactate dehydrogenase (LDH) to evaluate whether it could predict survival benefits of PPTR in mCRC patients.

## 2 Materials and methods

### 2.1 Patient selection

The research protocol of the study was approved by the Institutional Review Board (IRB) of Sir Run Run Shaw Hospital (SRRSH), Zhejiang University (reference No: 20040105-06). Written informed consent was obtained from all patients enrolled in this study. This was a single center, retrospective analysis of subjects at SRRSH in a 9-year period (2005-2013). Patients met the following criteria were included in the study:

(1) patients diagnosed as mCRC at first visit, between January 2005 and December 2013;
(2) patients with metastases that were considered unresectable by a multidisciplinary team;
(3) patients for whom follow-up information was available;
(4) patients for whom preoperative complete blood cell (CBC), biochemical indexes, and tumor markers were available.

Patients with the following conditions were excluded:

(1) obstruction, perforation, or bleeding at the first visit or had undergone an emergency operation;
(2) presence of other primary tumors;
(3) resectable metastases.

### 2.2 Blood sample analysis

Data of preoperative CBC, biochemical indexes, and tumor markers were retrospectively extracted from the patients’ medical records. All these blood samples had been obtained within 2 weeks before the surgery.

### 2.3 Evaluation of novel scoring system

We devised a scoring system with a combination of 4 prognostic factors, namely, NLR, CEA, CA19-9, and LDH (Table 1). The total score was 4. Patients were classified into 3 groups as follows: high score group (3 and 4), intermediate score group (1 and 2), and low score group (0).

**Table 1:**
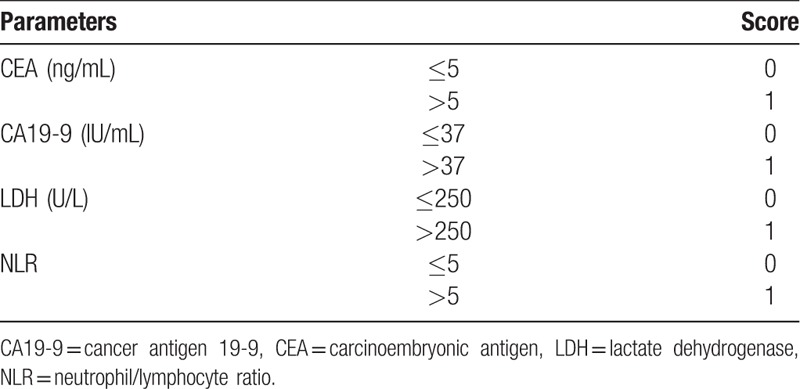
Scoring system to predict survival benefit of palliative primary tumor resection in metastatic colorectal cancer.

### 2.4 Statistical analysis

Patient characteristics and outcomes were summarized by descriptive statistics, such as the median or mean, inter-quartile interval [CI], and range. *χ*^*2*^ test was used to evaluate the differences between the PPTR and Non-PPTR groups in the baseline characteristics. OS curves and survival outcomes difference between the groups were made by the Kaplan–Meier and log rank test (Mantel–Cox), respectively. Patients were excluded from the study at the time of their last follow-up if they had died, if there were no available data, or if they were lost to follow-up. Associations between OS, clinical variables, and PPTR were identified and quantified by univariate and multivariate Cox proportional hazards regression analysis. *P* values < .05 were considered statistically significant. SPSS version 22.0 was used for statistical analyses.

## 3 Results

### 3.1 Patient characteristics

**Figure 1:**
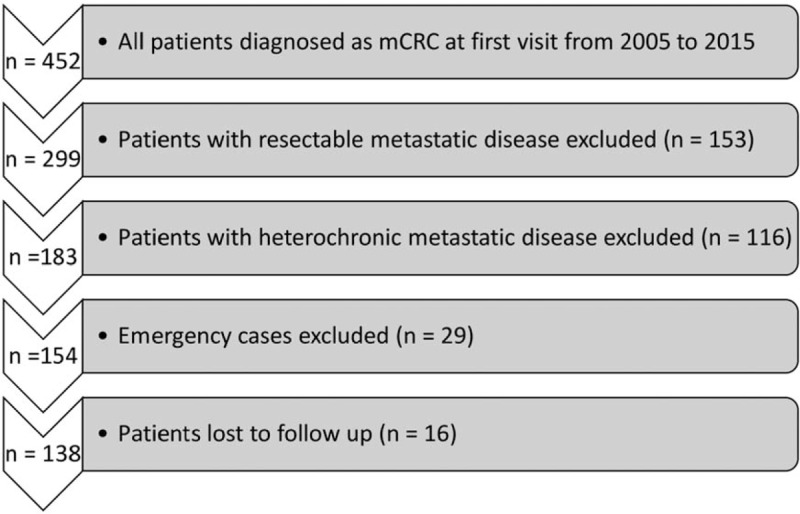
Flow diagram of patients included and excluded in this study. mCRC = metastatic colorectal cancer.

Overall, there were 138 patients involved in this study after excluding patients with resectable metastatic diseases (135) and heterochronic metastatic disease (116). Additionally, 29 patients who underwent emergency surgery and 16 patients who were lost to follow-up were excluded (Fig. 1).

The baseline characteristics of the 138 synchronous mCRC patients have been shown in Table 2: 103 patients underwent palliative primary tumor resection (PPTR) and 35 patients did not undergo resection.

**Table 2:**
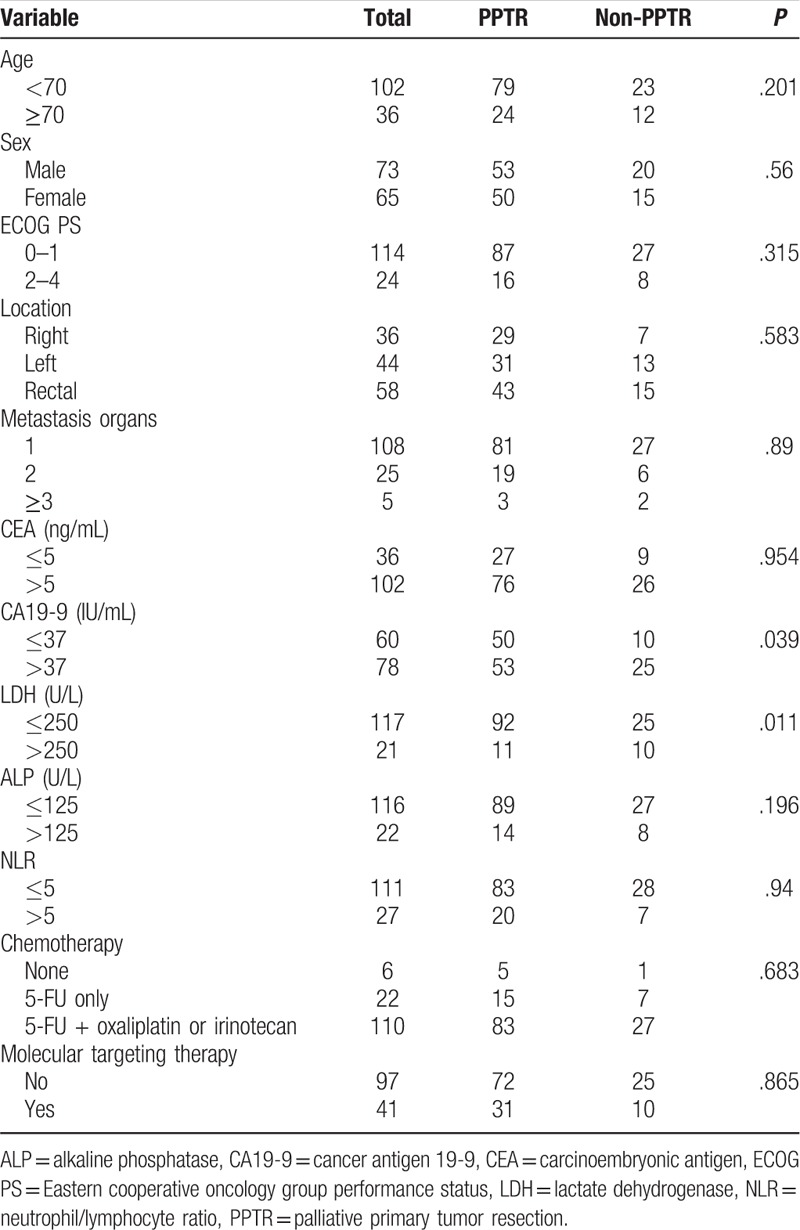
Characteristics of the 138 colorectal carcinoma patients with synchronous unresectable metastasis.

The age range of the synchronous mCRC patients with PPTR ranged from 21 to 84 years (median 60), while those without initial resection were from 43 to 86 years (median 67). There was no significant relevance between the primary tumor resection and age, Eastern cooperative oncology group performance status, gender, primary tumor site, metastasis organs, CEA, alkaline phosphatase (ALP), or NLR. However, patients with low levels of CA19-9 (*P* = .039) or LDH (*P* = .011) were more likely to undergo an operation.

### 3.2 Effect of PPTR on survival

**Figure 2:**
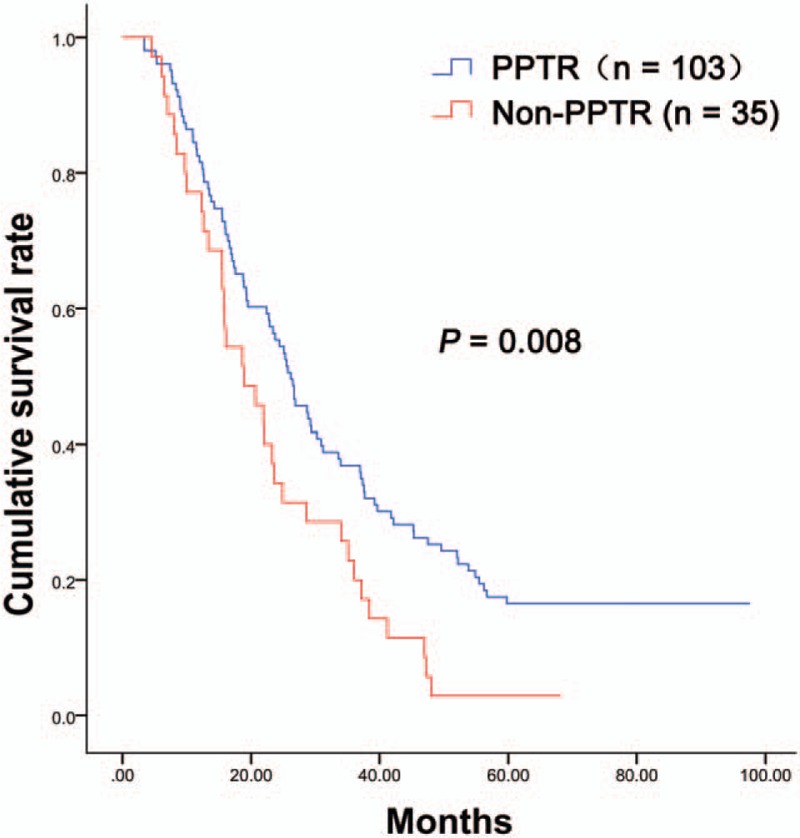
The 5-year survival of the PPTR (n = 103) and Non-PPTR (n = 35) groups. PPTR = palliative primary tumor resection.

There was a significantly longer OS in the PPTR group (26.2 months, 14.3–49.6 months) than that in the Non-PPTR group (18.9 months, 12.3–35.1 months) (*P* = .008) (Fig. 2). Multivariate analysis identified PPTR as an independent good prognostic factor (*P* = .027) (Table 3).

**Table 3:**
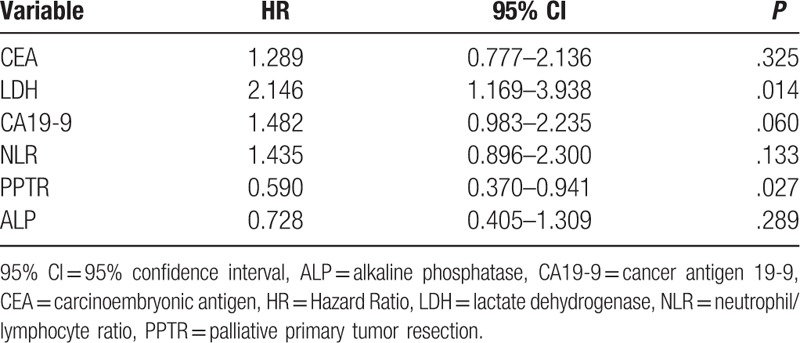
Multivariate analysis of prognostic factors in patients with synchronous metastatic colorectal cancer (n = 138).

### 3.3 Screening patients for PPTR using the novel scoring system

We then used univariate and multivariate analyses to analyze various clinical factors that might be used to predict survival. Multivariate analysis revealed that PPTR was an independent prognostic factor with a better survival (*P* = .027) (Table 3). Moreover, in the PPTR subgroup, except for the level of ALP (*P* = .123), the high levels of CEA, CA19-9, LDH, and NLR (>5) predicted poorer survival (Table 4). Based on these results, a scoring system was established, which was divided into 0, 1, 2, 3, and 4 points. For convenience of grouping, it was divided into low- (0), intermediate- (1–2), and high- (3–4) score groups.

**Table 4:**
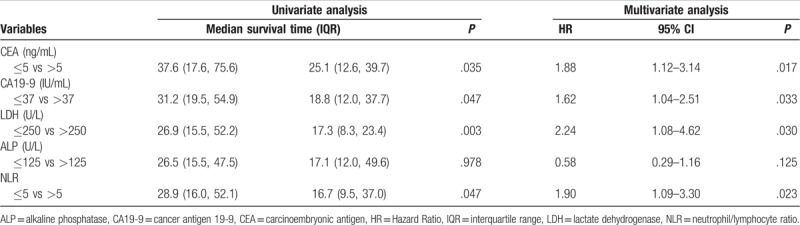
Prognostic factor results in colorectal carcinoma patients with synchronous unresectable metastasis who underwent palliative primary tumor resection (n = 103).

**Figure 3:**
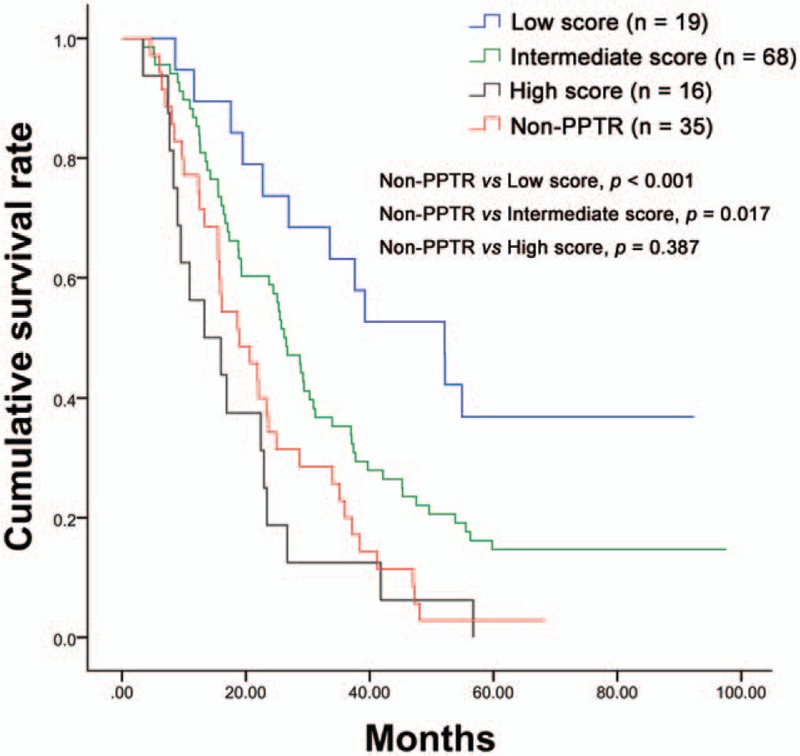
The 5-year survival of the mCRC patients who underwent PPTR subdivided into low-, intermediate-, and high-score groups according to the scoring system, and the Non-PPTR group. The median survival of the low- and intermediate-score groups were significantly (*P* < .001, *P* = .017 respectively) better than that of the Non-PPTR group. However, the high-score group showed no difference from the Non-PPTR group (*P* = .387). mCRC = metastatic colorectal cancer, PPTR = palliative primary tumor resection.

Consequently, patients who underwent PPTR with low scores (18.4%) had significantly (*P* < .001) longer survival (52.1 months, 22.7–70.8 months) than those in the no operation group (18.9 months, 12.3–35.1 months). Moreover, the OS (26.2 months, 15.5–45.2 months) of the intermediate group (66.0%) was 7.3 months, significantly (*P* = .017) longer than that of the Non-PPTR group. However, the PPTR group patients with a high score (13.3 months, 8.3–22.9 months) had significantly worse survival than those in the low or intermediate score group (*P* < .001, *P* = .002, respectively), and was 5.6 months shorter than that of the Non-PPTR group, though the difference was not significant (*P* = .387). (Fig. 3)

## 4 Discussion

In this study, we assessed the combined value of NLR, CEA, CA19-9, and LDH as a better predictor of patient survival. The patients who underwent PPTR from the low score or intermediate score group had a significantly longer survival than those from the no operation group; a low score was especially found to be a strong indication for PPTR with a 33.2 months longer survival time. However, the high score group showed no survival benefit from PPTR.

Primary tumor resection in asymptomatic CRC patients with incurable metastasis remains controversial, although it has been advocated to treat symptoms such as perforation, bleeding, or obstruction. Surgical cytoreduction has shown survival benefits in certain other types of cancers, such as, advanced renal^[18,19]^ and ovarian^[20,21]^ cancers. However, it is not clear whether this theory can be applied directly to CRC. Some retrospective series ^[22–24]^ or meta-analysis^[25,26]^ studies have shown that PPTR in patients with unresectable mCRC could prolong survival. A meta-analysis of 148,151 patients by Nische et al^[27]^ revealed significantly improved survival and reduced 30-day mortality in the resection group and no significant difference in the morbidity.

Conversely, some studies have shown the opposite results, because the development of chemotherapy and targeted drugs has significantly increased patient survival.^[6–9]^ Moreover, PPTR interferes with timely initiation of cytotoxic chemotherapy^[28]^ or even precludes chemotherapy administration because of complications.^[29]^ It also contributes to a 20% to 35% morbidity rate and a 6% to 10% mortality rate.^[23,29–31]^ Furthermore, some studies have found that increased metastatic burden is a possibility following surgery owing to flare-up metastatic angiogenesis and immune response alteration.^[32–34]^

Currently, all the evidence is from retrospective data, with a low level of evidence for the high rate of high-risk bias and heterogeneity. In the non-randomized study design, selection biases are inevitable in the raw analysis of survival, because the decision whether a patient should undergo PPTR is made case-by-case. In patients with unresectable mCRC, acceptance of PPTR is a complicated and personalized decision, and therefore, it is extremely difficult to design an RCT study. Thus, a systematic and practicable scoring system is imperative to select patients who may benefit from PPTR.

It has been confirmed by a one-pool study^[17]^ that included 1613 consecutive patients with colorectal liver metastasis that serum CA19-9 level <37 u/mL and CEA level <5 ng/mL were predictors of a favorable result. In a variety of cancers including mCRC, elevated LDH levels have been proved to be associated with poor prognosis.^[35,36]^ Elevated LDH levels in mCRC accelerated the growth kinetics by activating hypoxia-inducible factor-related genes in aggressive tumor phenotypes.^[37]^ However, as LDH levels may be influenced by systemic infection, attention should be given to inflammatory markers.

NLR is one of the inflammatory biomarkers that has been proved an optimal predictor in pancreatic, hepatocellular, esophageal, lung, and CRC.^[11,38–40]^ NLR reflects the host systemic inflammatory/immune response. Neutrophils play an important role in promoting vascularization and proliferation in tumor tissue, by producing pro-angiogenic chemokines, ligands, and other factors.^[41,42]^ Neutrophils also promote the adhesion of circulating tumor cells and the end-organ, which increases the chances of metastatic seeding.^[43,44]^ Lymphocytes therefore play an important role in tumor inhibition and lymphopenia is an indicator of poor prognosis in cancer patients.^[45,46]^ The function of lymphocytes is to produce cytokines in tumor cells and suppress cytotoxic cell death. Tumor-infiltrating lymphocytes (TIL) are good prognostic indicators for various cancers, possibly because of TIL-induced anti-tumor activity and inhibition of angiogenesis.^[47,48]^

In this study, the parameters included in this novel scoring system are all standardized widely available assays that are cheap and easy to measure. This scoring system would be helpful to patients with unresectable mCRC in choosing the optimal treatment plan.

## 5 Conclusion

In this study, we developed a scoring system integrating the values of CEA, NLR, LDH, and CA19-9 to predict survival in patients with unresectable mCRC who underwent PPTR. This novel scoring system based on these 4 assays not only classified mCRC patients into 3 independent risk groups before surgery but also helped predict postoperative survival of these patients. However, further RCT studies are needed to prove the clinical value of this scoring system.

## Author contributions

**Conceptualization:** Gaoyang Cao, Wei Zhou, Zhangfa Song.

**Data curation:** Gaoyang Cao, Wei Zhou, Fei Wang, Guolin Zhang, Zhangfa Song.

**Formal analysis:** Gaoyang Cao, Wei Zhou, Engeng Chen, Zhangfa Song.

**Funding acquisition:** Gaoyang Cao, Wei Zhou, Jianbin Xu, Zhangfa Song.

**Investigation:** Gaoyang Cao, Wei Zhou, Engeng Chen, Fei Wang, Guolin Zhang, Zhangfa Song.

**Methodology:** Gaoyang Cao, Wei Zhou, Engeng Chen, Fei Wang, Jianbin Xu, Zhangfa Song.

**Resources:** Li Chen, Wei Zhao, Wei Zhang.

**Software:** Engeng Chen, Fei Wang, Min Chen, Jianbin Xu, Wei Zhang.

**Supervision:** Jianbin Xu, Wei Zhang, Xuefeng Huang, Zhangfa Song.

**Validation:** Li Chen, Min Chen, Wei Zhang.

**Visualization:** Wei Zhao.

**Writing – original draft:** Gaoyang Cao, Wei Zhou.

**Writing – review & editing:** Gaoyang Cao, Wei Zhou, Xuefeng Huang, Zhangfa Song.

Gaoyang Cao orcid: 0000-0003-0664-538X.
